# Growth, ionic homeostasis, and physiological responses of cotton under different salt and alkali stresses

**DOI:** 10.1038/s41598-020-79045-z

**Published:** 2020-12-14

**Authors:** Huijuan Guo, Zhijie Huang, Meiqi Li, Zhenan Hou

**Affiliations:** Department of Resources and Environmental Science, Agriculture College, Shihezi University, 425# Box, Shihezi, 832003 Xinjiang People’s Republic of China

**Keywords:** Plant sciences, Plant stress responses

## Abstract

To better understand the mechanism of salt tolerance, we analyzed cotton growth and the ionomes in different tissues under different types of salt–alkali stress. Cotton was exposed to the soil salt and alkali stresses, NaCl, Na_2_SO_4_, and Na_2_CO_3_ + NaHCO_3_, in a pot study. Salt and alkali stress significantly inhibited cotton growth, significantly reduced root length, surface area, and volume, and significantly increased relative electrical conductivity (REC) and malondialdehyde (MDA) content but also significantly increased antioxidant enzyme activities, and proline (Pro) content. The REC in leaves was higher under salt stress than under alkali stress, but the effects on Pro were in the order Na_2_CO_3_ + NaHCO_3_ > NaCl > Na_2_SO_4_. Principal component analysis showed a significant difference in ion composition under the different types of salt–alkali stress. Under the three types of salt–alkali stress, concentrations of Na and Mo increased significantly in different organs of cotton plants. Under NaCl stress, the absorption of Ca was inhibited, the transport capacity of P, Mg, and Cu was reduced, and the ion balance was maintained by promoting the uptake and transport of Zn, Mn, Al, and Mo. Under Na_2_SO_4_ stress, the absorption of P and Ca was inhibited, the transport capacity of Mg, B, and Cu was reduced, and the ion balance was maintained by promoting the uptake and transport of S, Zn, Fe, Mo, Al, and Co. Under Na_2_CO_3_ + NaHCO_3_ stress, the absorption of P and S was inhibited, the transport capacity of Mg and B was reduced, but that of Al and Fe increased, and the ion balance was maintained by promoting the uptake and transport of Mn, Mo, Ni, and Co. The relative expression of *GhSOS1* and *GhNHX1* in leaves increased significantly under salt stress but decreased under alkali stress. These results suggest that cotton is well-adapted to salt–alkali stress via the antioxidant enzyme system, adjustment of osmotic substances, and reconstruction of ionic equilibrium; neutral salt stress primarily disrupts the ion balance, whereas alkali stress decreases the ability to regulate Na and inhibits the absorption of mineral elements, as well as disrupts the ion balance; and the changes in the expression of salt tolerance-related genes may partially explain the accumulation of Na ions in cotton under salt–alkali stress.

## Introduction

Soil salinization is a global ecological problem that threatens the environment and the development of sustainable agriculture^1^. Cotton is a “pioneer crop” used to develop and use salt–alkali soil and is also a model plant used to study the mechanism of salt tolerance because of its relatively high salt tolerance. However, salt–alkali stress still greatly affects cotton growth^2^. With the increasing change in the global climate and the worsening of soil salinization, it is urgent that we improve salt tolerance in plants^3^. There are different types of soil salinization, with neutral and alkaline salts causing two distinct types of salt stress, to which crops have different responses and salt-tolerance mechanisms^4^. Salt stress occurs with neutral salts (NaCl and Na_2_SO_4_), with osmotic stress and ion toxicity the main effects on plants^5^. Alkali stress occurs with alkaline salts (NaHCO_3_ and Na_2_CO_3_)^6^, mainly because the high pH affects plant growth and disrupts the ion balance^7^. The risk of salt–alkali stress may be greater than that of neutral salt stress^8^. However, few studies have examined the effects of different types of salt stress, even though our understanding of the salt-tolerance mechanisms of crops under different salt–alkali stresses needs to be improved^9,10^.

The salt-tolerance mechanism of cotton has been widely studied. Under salt–alkali stress, the morphological index and root development during cotton growth can most intuitively reflect the stress status of cotton. According to Wang et al.^11^ and Chachar et al.^12^, cotton growth is significantly inhibited, seedling biomass is significantly reduced, and root development is inhibited under salt–alkali stress. Furthermore, salt–alkali stress causes ion toxicity in plants, as a result of imbalances disrupting ion homeostasis. The reconstruction of ion homeostasis under salt–alkali stress is one important mechanism by which plants improve their salt–alkali tolerance^13^. Ionomics is a new approach to study the response of plants to salt–alkali stress, and the mineral nutrient and trace element ionome of plants can be used to characterize the inorganic components of cells and biological systems^14^. Mineral elements guarantee the growth of crops^15^. However, primarily because excess salt ions in soil cause competition among ions for absorption in cotton under salt–alkali stress, the absorption and transport of mineral elements are substantially affected, resulting in mineral nutrient stress and imbalance in crop ion homeostasis. Salt stress not only inhibits the uptake of macro elements (N, P, K, Ca, Mg, S) by crops but also limits the absorption of trace elements (Fe, Cu, Zn, Mn, and B, among others)^16^. In addition to being nutrients for crop growth, these mineral elements also participate in various physiological metabolic processes that directly or indirectly affect the salt tolerance of crops.

Although each element has a unique physiological function in crop growth, the primary function of each is to maintain intracellular ionic homeostasis. Maintaining intracellular ionic homeostasis is an important mechanism by which crops adapt to salt stress, and all the physiological activities related to salt tolerance in crops are aimed at maintaining ionic homeostasis. Therefore, to reveal the mechanism of salt tolerance in crops, it is important to examine the mechanisms that maintain ionic homeostasis during the absorption of mineral nutrients by plants under salt stress. The absorption of ions by cotton under salt stress and the use of mineral elements to improve the salt tolerance of cotton have been investigated^17,18^. However, the focus of most previous studies has been on the effects of salt stress on one or more mineral elements, and the responses of other elements and their interactions with salt stress have not been fully elucidated^16^. Therefore, our understanding of the relationship between the mechanism of salt-tolerance and the ionome remains fragmented and incomplete.

Environmental factors can also influence ion homeostasis by regulating associated genes. Two key salt-tolerance genes are *SOS1* and *NHX1*^19,20^. The SOS pathway is involved in the maintenance of ionic homeostasis, and *NHX1* participates in Na^+^ transport and compartmentalization, maintains osmotic balance, and reduces cytosolic Na^+^ concentration^21^. Therefore, the analysis of the expression of Na^+^ transport-related genes can help to explain the changes in ion homeostasis and reveal the salt-tolerance mechanism in cotton under salt–alkali stress.

The salt tolerance of crops involves a variety of defense mechanisms, including maintaining ionic homeostasis and osmotic equilibrium and scavenging reactive oxygen species (ROS)^22,23^. In ionomics, high-throughput analysis (ICP-MS) is used to quantitatively study the ionome characteristics of an organism, providing an important approach to understand the element–element and element–environment interactions as well as the physiological and biochemical functions of elements^24^. Because salt–alkali stress is one of the most serious abiotic stress factors limiting crop production, an in-depth understanding of the responses of ionomes under salt–alkali stress remains essential to understand the mechanism of ionic homeostasis in cotton. In addition, as understanding increases, we can provide a theoretical basis to improve the salinity and alkalinity tolerance of cotton by ionic regulation and rational fertilization in salt–alkali soil, as well as provide a reference for the breeding of salt-tolerant cotton cultivars.

In this study, we examined the effects of different types of salt–alkali stress on (1) the biomass of cotton plants and the morphological characters of root length, surface area, and volume; (2) the physiological indices of salt tolerance (REC, MDA, Pro), the biochemical indices (activities of superoxide dismutase (SOD), peroxidase (POD), catalase (CAT)), and the mechanisms of organic osmotic regulation and enzyme protection; (3) the ionomic responses and distributions of the main mineral elements in cotton plants, as well as the correlations between Na and other elements; and (4) the expression of Na^+^ transport-related genes involved in ionic homeostasis in cotton.

## Results

### Changes in cotton biomass and root morphology after salt and alkali stresses

**Figure 1:**
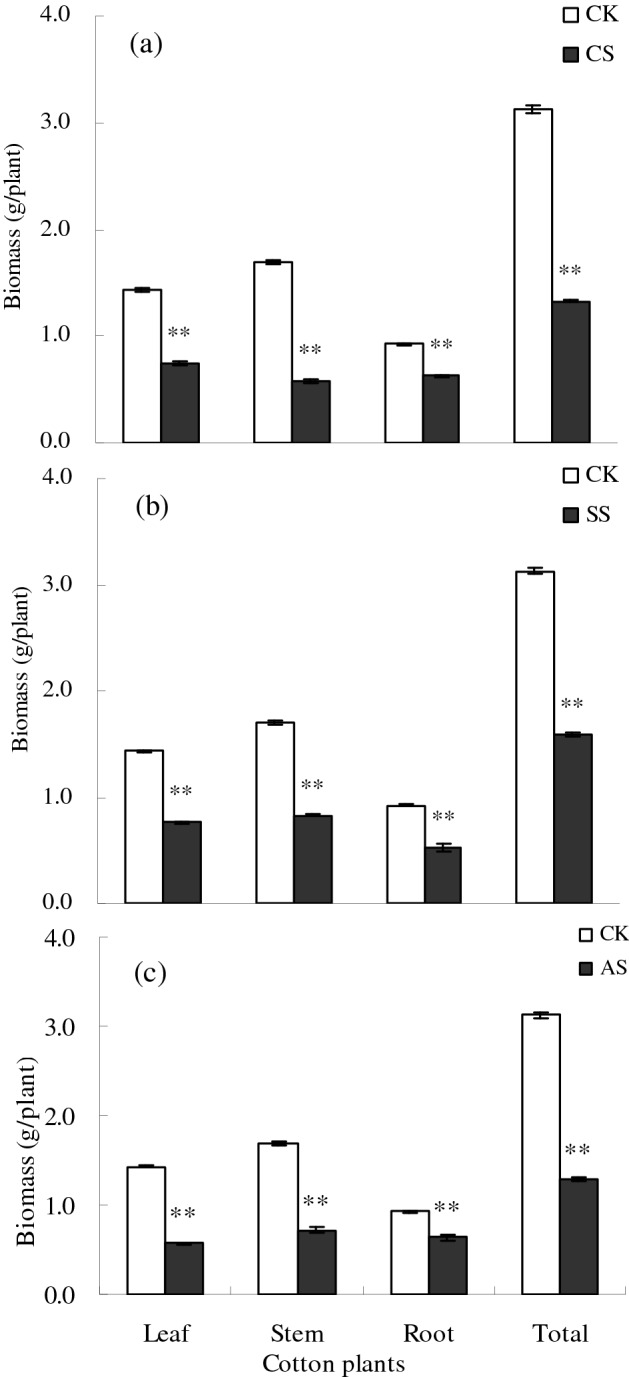
Leaf, stem, root, and total biomass (g/plant) of cotton plants under different types of salt–alkali stress: (**a**) NaCl (CS), (**b**) Na_2_SO_4_ (SS), and (**c**) Na_2_CO_3_ + NaHCO_3_ (AS). Columns with bars represent the mean ± standard error (*n* = 3). Asterisks indicate a significant difference between the control (CK) and the salt–alkali stress (**p < 0.01).

Cotton biomass decreased significantly under salt–alkali stress (Fig. 1). Compared with the CK, total cotton biomass decreased by 57.55% in the CS treatment, by 49.19% in the SS treatment, and by 58.61% in the AS treatment. Compared with the CK, the biomass of the root, stem, and leaves was significantly lower by 47.98%, 65.64%, and 32.07%, respectively, in the CS treatment (Fig. 1a), by 46.86%, 51.17%, and 43.18%, respectively, in the SS treatment (Fig. 1b), and by 59.93%, 57.50%, and 31.17%, respectively, in the AS treatment (Fig. 1c).

**Figure 2:**
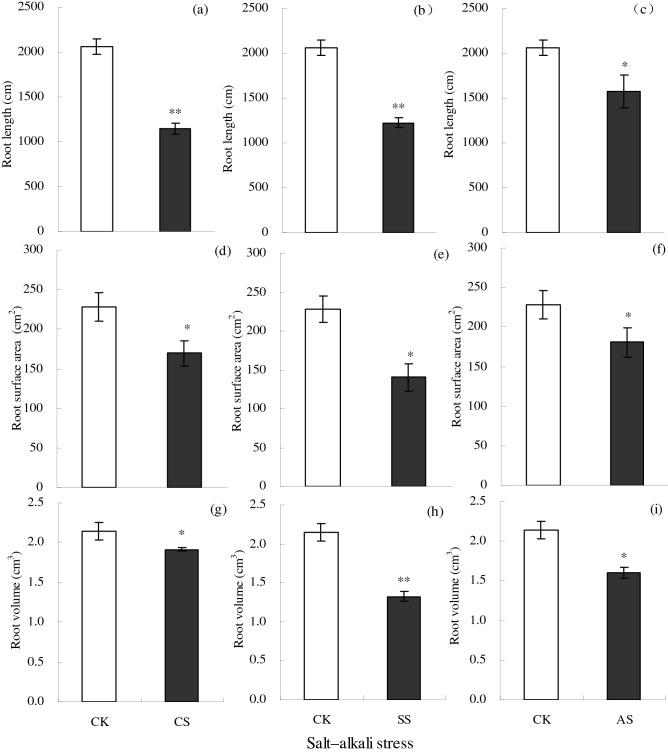
Root length (cm), surface area (cm^2^), and volume (cm^3^) of cotton plants under different types of salt–alkali stress (NaCl (CS), Na_2_SO_4_ (SS), and Na_2_CO_3_ + NaHCO_3_ (AS)). Columns with bars represent the mean ± standard error (*n* = 3). Asterisks indicate a significant difference between the control (CK) and the salt–alkali stress (*p < 0.05; **p < 0.01). (**a**–**c**) indicate the root length in NaCl, Na_2_SO_4_, and Na_2_CO_3_ + NaHCO_3_ stress, respectively. (**d**–**f**) indicate the surface area in NaCl, Na_2_SO_4_, and Na_2_CO_3_ + NaHCO_3_ stress, respectively. (**g**–**i**) indicate the root volume in NaCl, Na_2_SO_4_, and Na_2_CO_3_ + NaHCO_3_ stress, respectively.

Salt–alkali stress significantly decreased root length, surface area, and volume (Fig. 2). Compared with the CK, root length, surface area, and volume decreased significantly by 44.33%, 25.62%, and 10.80%, respectively, in the CS treatment, by 40.53%, 38.49%, and 38.40%, respectively, in the SS treatment; and by 23.63%, 20.85%, and 25.28%, respectively, in the AS treatment.

### Changes in cotton physiological response after salt and alkali stresses

**Figure 3:**
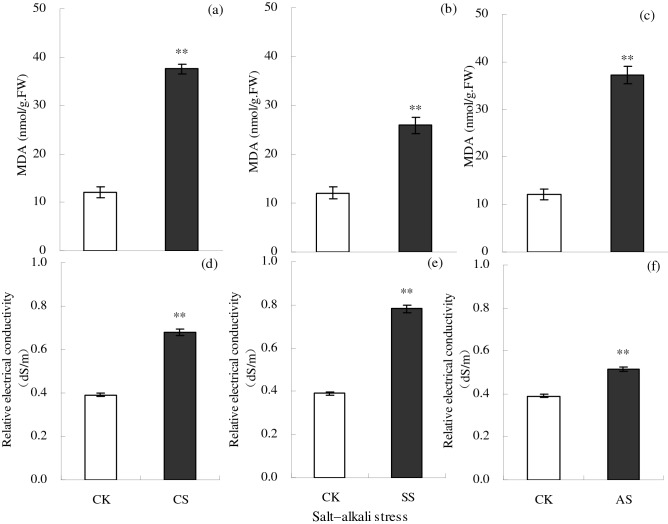
Malondialdehyde (MDA) content (nmol/g fresh weight (FW)) and relative electrical conductivity (REC) (dS/m) in cotton leaves under different types of salt–alkali stress (NaCl (CS), Na_2_SO_4_ (SS), and Na_2_CO_3_ + NaHCO_3_ (AS)). Columns with bars represent the mean ± standard error (*n* = 3). Asterisks indicate a significant difference between the control (CK) and the salt–alkali stress (**p < 0.01). (**a**–**c**) indicate the MDA content in cotton leaves under NaCl, Na_2_SO_4_, and Na_2_CO_3_ + NaHCO_3_ stress, respectively. (**d**–**f**) indicate the REC in cotton leaves under NaCl, Na_2_SO_4_, and Na_2_CO_3_ + NaHCO_3_ stress, respectively.

The MDA content and REC of leaves increased significantly under salt–alkali stress (Fig. 3). Compared with the CK, the MDA content in the leaves increased significantly by 211.72% in the CS treatment, by 114.48% in the SS treatment, and by 208.28% in the AS treatment. Similarly, compared with the CK, the REC in leaves increased significantly by 74.06% in the CS treatment, by 99.83% in the SS treatment, and by 31.54% in the AS treatment.

**Figure 4:**
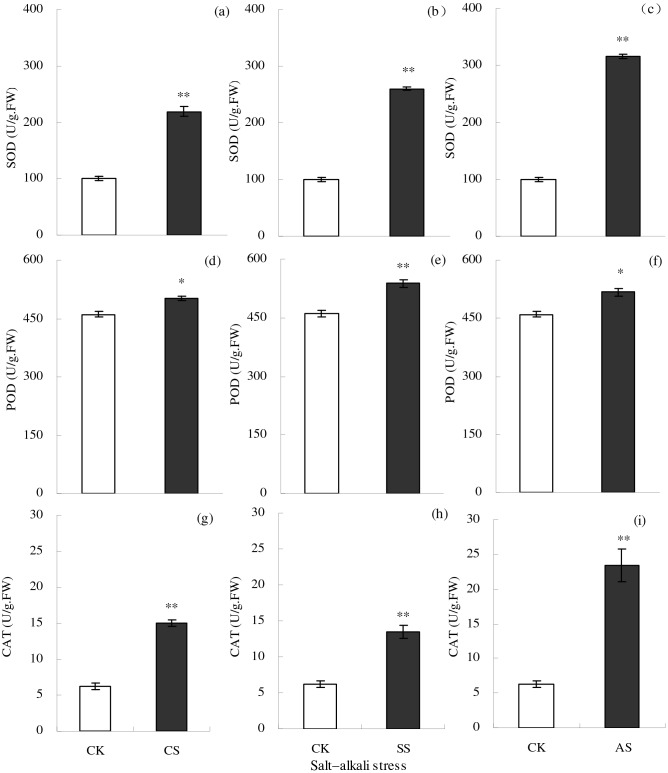
Activities (U/g fresh weight (FW)) of the antioxidant enzymes superoxide dismutase (SOD), peroxidase (POD), and catalase (CAT) in cotton leaves under different types of salt–alkali stress (NaCl (CS), Na_2_SO_4_ (SS), and Na_2_CO_3_ + NaHCO_3_ (AS)). Columns with bars represent the mean ± standard error (*n* = 3). Asterisks indicate a significant difference between the control (CK) and the salt–alkali stress (*p < 0.05; **p < 0.01). (**a**–**c**) indicate the SOD in cotton leaves under NaCl, Na_2_SO_4_, and Na_2_CO_3_ + NaHCO_3_ stress, respectively. (**d**–**f**) indicate the POD in cotton leaves under NaCl, Na_2_SO_4_, and Na_2_CO_3_ + NaHCO_3_ stress, respectively. (**g**–**i**) indicate the CAT in cotton leaves under NaCl, Na_2_SO_4_, and Na_2_CO_3_ + NaHCO_3_ stress, respectively.

Salt–alkali stress significantly increased the activities of SOD, POD, and CAT in leaves (Fig. 4). Compared with the CK, the SOD activity increased significantly by 118.89% in the CS treatment, by 159.92% in the SS treatment, and by 215.29% in the AS treatment; the POD activity increased significantly by 8.98% in the CS treatment, by 16.80% in the SS treatment, and by 12.38% in the AS treatment; and the CAT activity increased significantly by 139.78% in the CS treatment, by 116.70% in the SS treatment, and by 275.91% in the AS treatment.

**Figure 5:**
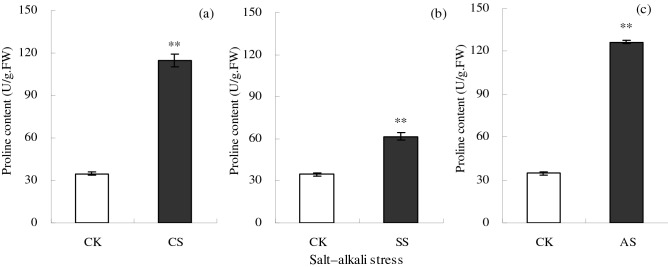
Proline content (U/g fresh weight (FW)) in cotton leaves under different types of salt–alkali stress (NaCl (CS), Na_2_SO_4_ (SS), and Na_2_CO_3_ + NaHCO_3_ (AS)). Columns with bars represent the mean ± standard error (*n* = 3). Asterisks indicate a significant difference between the control (CK) and the salt–alkali stress (**p < 0.01). (**a**–**c**) indicate the Proline content in cotton leaves under NaCl, Na_2_SO_4_, and Na_2_CO_3_ + NaHCO_3_ stress, respectively.

Salt–alkali stress significantly increased the Pro content in cotton leaves (Fig. 5). Compared with the CK, the proline content in the leaves increased significantly by 230.07% in the CS treatment, by 77.91% in the SS treatment, and by 264.19% in the AS treatment.

### Changes in cotton tissue ionomes after salt and alkali stresses

**Figure 6:**
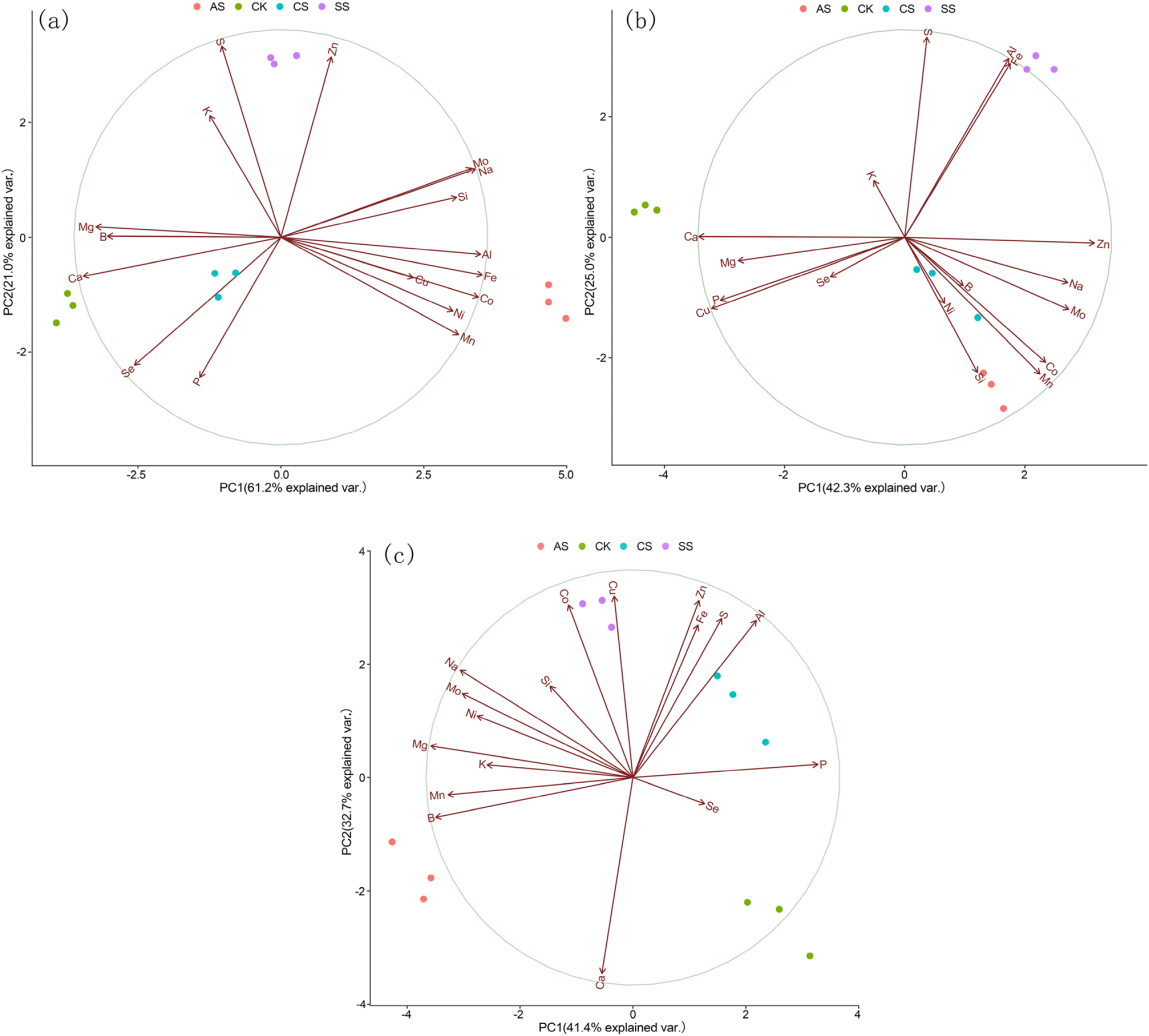
Principal component analysis (PCA) of tissue ionome variation in cotton under different types of salt–alkali stress (Control (CK), NaCl (CS), Na_2_SO_4_ (SS), and Na_2_CO_3_ + NaHCO_3_ (AS)), with the loadings of mineral elements to the PC1 and PC2. (**a**) Leaf ionome variation among samples and the loadings of elements to the PC1 and PC2; (**b**) Stem ionome variation among samples and the loadings of elements to the PC1 and PC2; (**c**) Root ionome variation among samples and the loadings of elements to the PC1 and PC2.

To demonstrate the effect of salt–alkali stress on element distribution in cotton, we analyzed the concentrations of the ions Na, P, K, Ca, Mg, S, Fe, Mn, Zn, Cu, B, Mo, Ni, Co, Al, Si, and Se in the leaf, stem, and root under different types of salt–alkali stress. In the PCAs, the leaf (Fig. 6a), stem (Fig. 6b), and root (Fig. 6c) ionomes were separated on the basis of different types of salt–alkali stress. The different types of salt–alkali stress were well separated on the first principal component, accounting for 61.2% of the total variation in leaves, 42.3% of that in stems, and 41.4% of that in roots. The major elements that contributed to the PC1 were Na, Ca, Mn, Fe, Mo, Al, and Co in the leaf ionome; Na, P, Ca, Cu, Zn, and Mo in the stem ionome; and Na, P, Mg, Mn, Mo, and B in the root ionome. Leaf and stem ionome analyses demonstrated that the SS treatment could be clearly distinguished from the other salt–alkali stress treatments using the second principal component, which explained 21.0% (leaf) and 25.0% (stem) of the total coefficient of variation. The contribution of elements to the PC2 was dominated by S and Zn in the leaf ionome and by S, Al, and Fe in the stem ionome. In the root ionome, the PC2 clearly distinguished between the neutral salt (CS and SS) treatments and the alkaline salt (AS) treatment, explaining 32.7% of the total coefficient of variation. The elements Ca, Cu, Zn, and Co were the dominant contributors to the PC2 in the root ionome.

**Figure 7:**
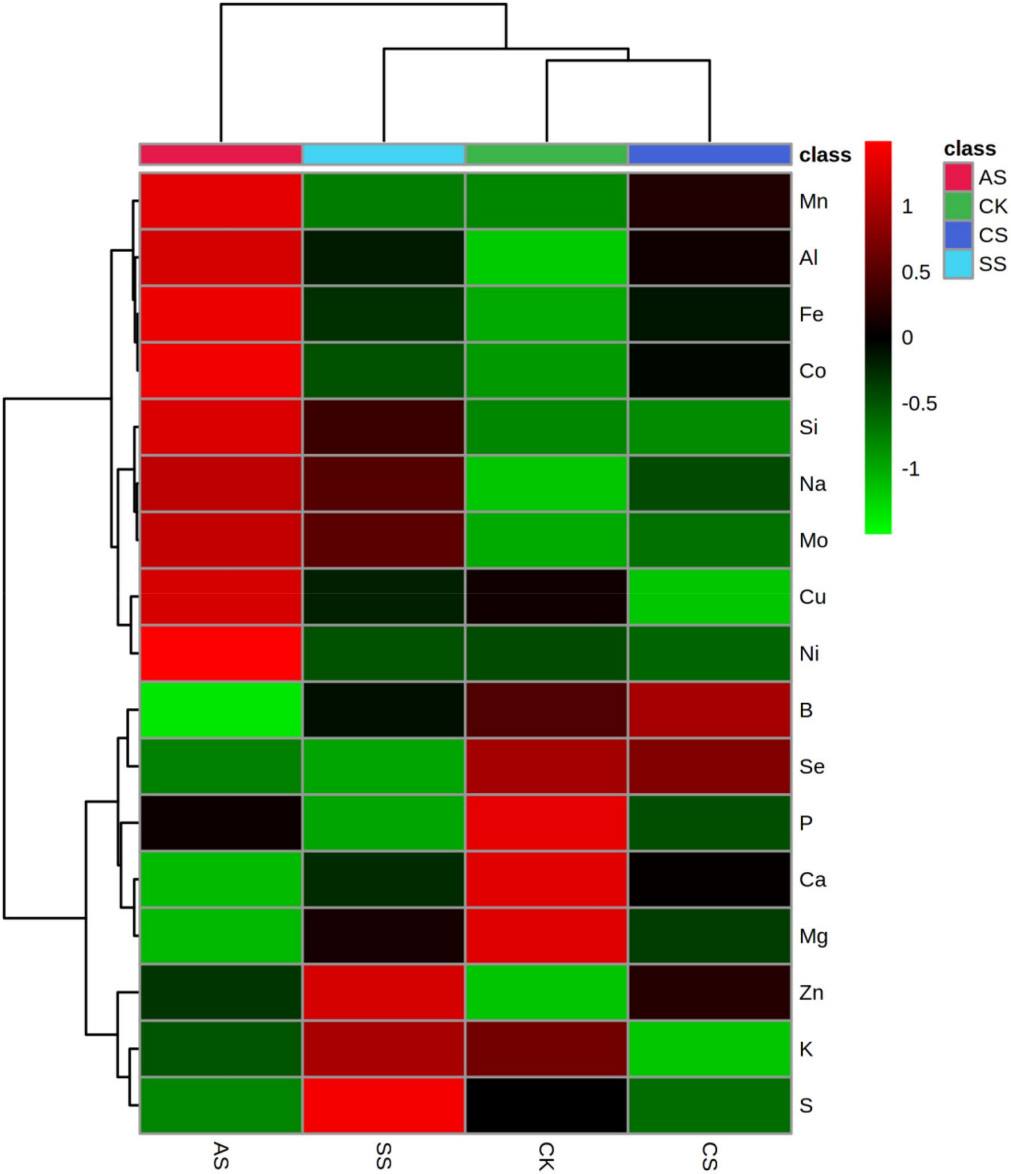
Hierarchical cluster analysis of the leaf ionomes in cotton plants under different types of salt–alkali stress (Control (CK), NaCl (CS), Na_2_SO_4_ (SS), and Na_2_CO_3_ + NaHCO_3_ (AS)). The relative values are indicated by color intensity in the legend in the upper right.

**Figure 8:**
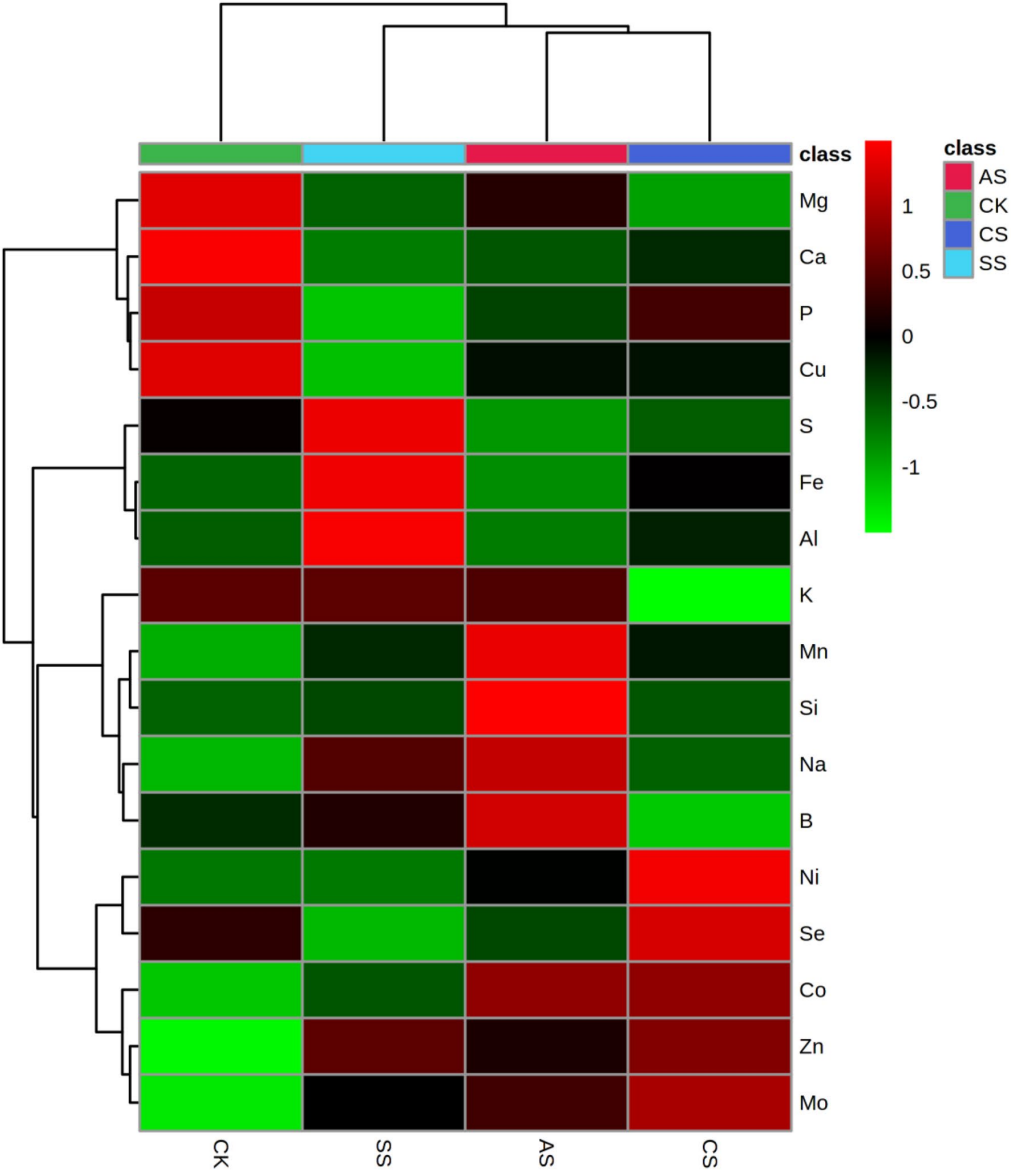
Hierarchical cluster analysis of the stem ionomes in cotton plants under different types of salt–alkali stress (Control (CK), NaCl (CS), Na_2_SO_4_ (SS), and Na_2_CO_3_ + NaHCO_3_ (AS)). The relative values are indicated by color intensity in the legend in the upper right.

**Figure 9:**
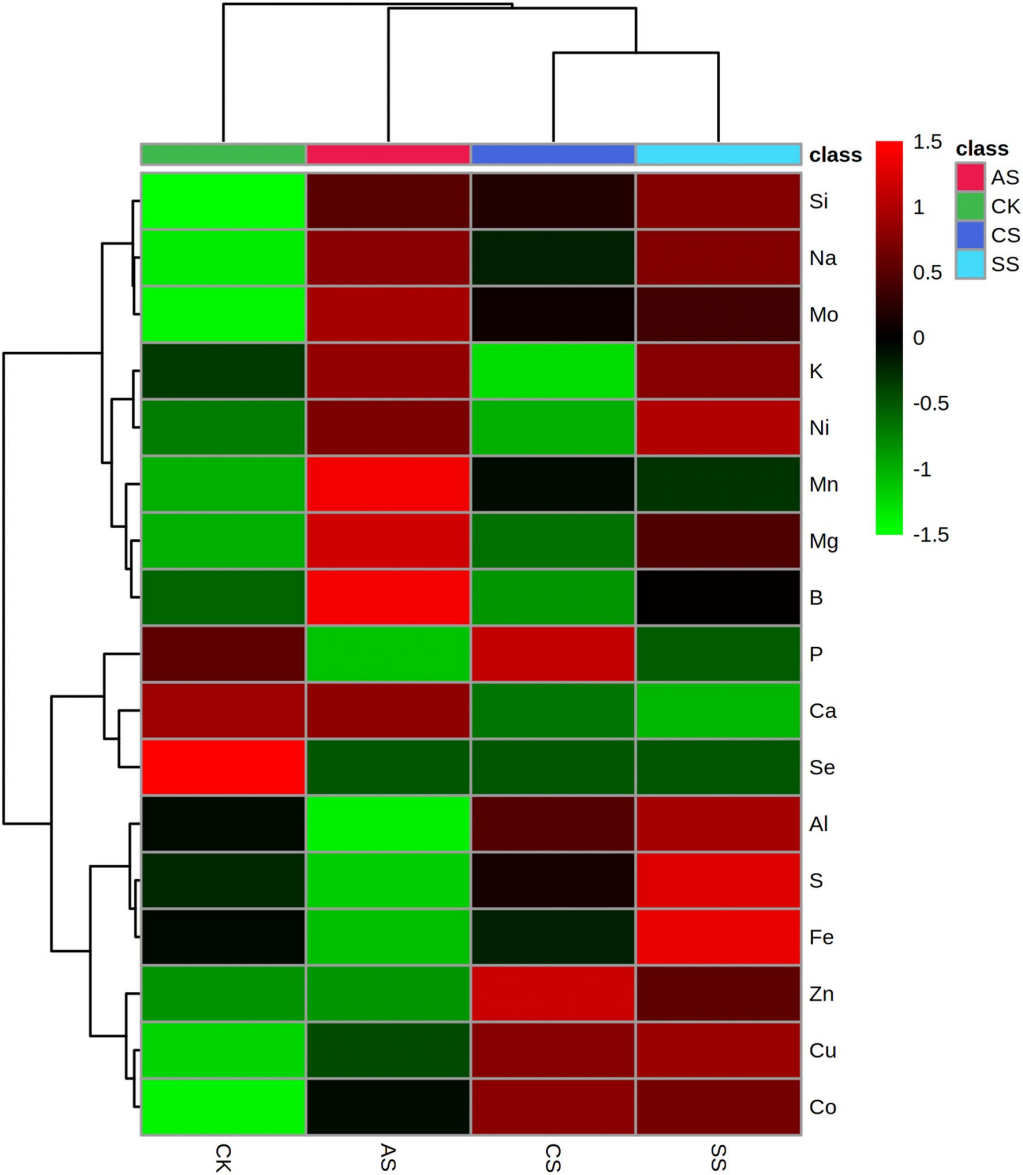
Hierarchical cluster analysis of the root ionomes in cotton plants under different types of salt–alkali stress (Control (CK), NaCl (CS), Na_2_SO_4_ (SS), and Na_2_CO_3_ + NaHCO_3_ (AS)). The relative values are indicated by color intensity in the legend in the upper right.

In the hierarchical cluster analysis, the leaf, stem, and root ionomes of cotton were also separated on the basis of different types of salt–alkali stress (Figs. 7, 8, and 9, respectively), indicating high similarity among the ionomes in the samples from each treatment. In leaves (Fig. 7, Tables S1–S3), compared with the CK, the concentrations of Na, Zn, Mn, Fe, B, Mo, Al, and Co increased significantly in the CS treatment; the concentrations of Na, S, Zn, Fe, Mo, Al, and Co increased significantly in the SS treatment; and the concentrations of Na, Fe, Mn, Zn, Cu, Mo, Ni, Co, Al, and Si increased significantly in the AS treatment. By contrast, the concentrations of P, K, Ca, Mg, S, and Cu decreased significantly in the CS treatment; the concentrations of P, Ca, Mg, B, Cu, and Se decreased significantly in the SS treatment; and the concentrations of P, K, Ca, Mg, S, B, and Se decreased significantly in the AS treatment.

In the stems (Fig. 8, Tables S4–S6), compared with the CK, the concentrations of Na, Zn, Mn, Fe, Mo, and Al increased significantly in the CS treatment; the concentrations of Na, S, Zn, Fe, Mo, Al, Mn, and Co increased significantly in the SS treatment; and the concentrations of Na, Mn, Zn, Mo, Ni, Co, and B increased significantly in the AS treatment. By contrast, the concentrations of B, Ca, Cu, P, K, Mg, and S decreased significantly in the CS treatment; the concentrations of P, Ca, Mg, and Cu decreased significantly in the SS treatment; and the concentrations of Al, Ca, Cu, Fe, Mg, P, and S decreased significantly in the AS treatment.

In the roots (Fig. 9, Tables S7–S9), compared with the CK, the concentrations of Na, P, Mg, Cu, Zn, Mn, Co, Mo, and Al increased significantly in the CS treatment; the concentrations of Na, Mg, S, Zn, Fe, Mo, B, Cu, Al, Mn, Ni, and Co increased significantly in the SS treatment; and the concentrations of Na, Mg, Mn, Mo, Ni, Co, and B increased significantly in the AS treatment. By contrast, the concentration of Ca decreased significantly in the CS treatment; the concentrations of P and Ca decreased significantly in the SS treatment; and the concentrations of Al, Fe, P, and S decreased significantly in the AS treatment.

### Changes in K/Na ratio after salt and alkali stresses

**Figure 10:**
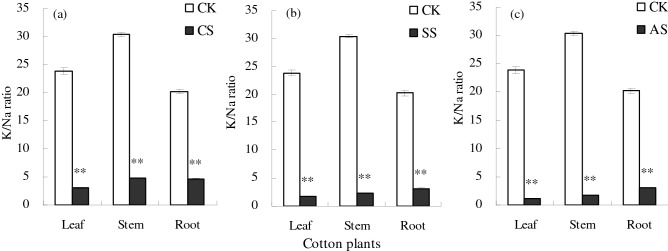
Leaf, stem, and root K/Na ratios of cotton plants under different types of salt–alkali stress: (**a**) NaCl (CS), (**b**) Na_2_SO_4_ (SS), and (**c**) Na_2_CO_3_ + NaHCO_3_ (AS). Columns with bars represent the mean ± standard error (*n* = 3). Asterisks indicate a significant difference between the control (CK) and the salt–alkali stress (**p < 0.01).

Salt–alkali stress significantly decreased the K/Na ratio in cotton plants (Fig. 10). Compared with the CK, the K/Na ratio in cotton leaves was significantly lower by 87.46% in the CS treatment, by 92.58% in the SS treatment, and by 95.20% in the AS treatment; the K/Na ratio in cotton stems was significantly lower by 84.16% in the CS treatment, by 92.22% in the SS treatment, and by 94.44% in the AS treatment; and the K/Na ratio in cotton roots was significantly lower by 76.92% in the CS treatment, by 84.43% in the SS treatment, and by 84.80% in the AS treatment.

### Relationship between Na element and other elements

**Figure 11:**
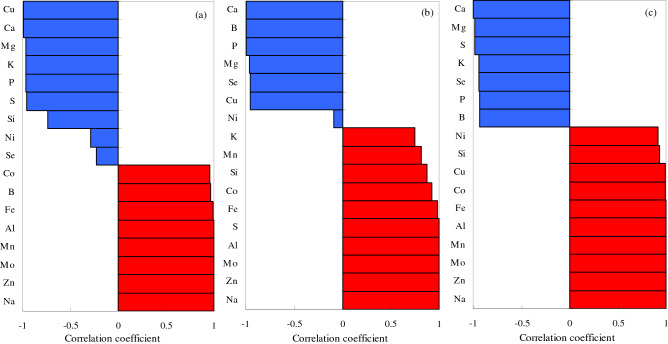
Pearson’s coefficients of correlation between the element Na and other elements in cotton leaves under different types of salt–alkali stress. (**a**) NaCl; (**b**) Na_2_SO_4_; and (**c**) Na_2_CO_3_ + NaHCO_3_. Blue, negative correlation; red, positive correlation.

**Figure 12:**
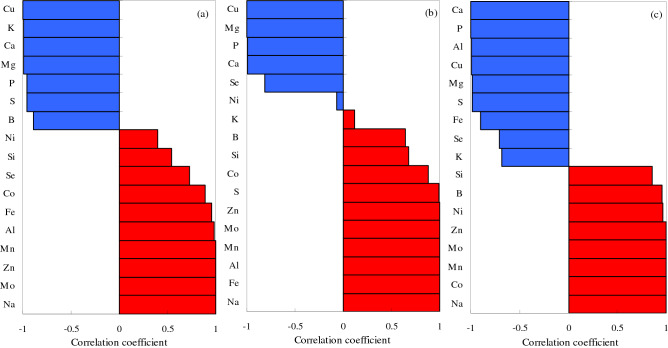
Pearson’s coefficients of correlation between the element Na and other elements in cotton stems under different types of salt–alkali stress. (**a**) NaCl; (**b**) Na_2_SO_4_; and (**c**) Na_2_CO_3_ + NaHCO_3_. Blue, negative correlation; red, positive correlation.

**Figure 13:**
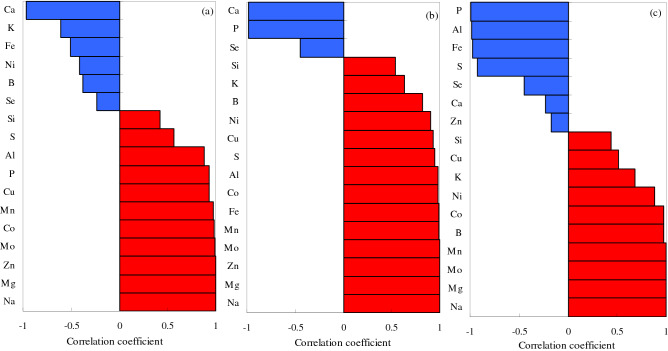
Pearson’s coefficients of correlation between the element Na and other elements in cotton roots under different types of salt–alkali stress. (**a**) NaCl; (**b**) Na_2_SO_4_; and (**c**) Na_2_CO_3_ + NaHCO_3_. Blue, negative correlation; red, positive correlation.

An excess of Na ions is the main cause of salt–alkali stress, and thus, it is essential to understand the correlations between Na and other elements. The correlations between Na and other elements in the leaf, stem, and root were analyzed under the different types of salt–alkali stress (Figs. 11, 12, 13). In the CS treatment, the Na levels in the leaves were significantly negatively correlated with seven elements (Cu, Ca, Mg, K, P, S, and Si) and significantly positively correlated with seven elements (Zn, Mo, Mn, Al, Fe, B, and Co), Ni and Se were negatively correlated with Na, but the correlations were not significant (Fig. 11a). In the SS treatment, the Na levels in the leaves were significantly negatively correlated with six elements (Ca, B, P, Mg, Se, and Cu) and significantly positively correlated with nine elements (Zn, Mo, Al, S, Fe, Co, Si, Mn, and K), but had a significantly negative correlation with Ni (Fig. 11b). In the AS treatment, the Na levels in the leaves were significantly negatively correlated with seven elements (Ca, Mg, S, K, Se, P, and B) and significantly positively correlated with nine elements (Zn, Mo, Mn, Al, Fe, Co, Cu, Si, and Ni), but had a significantly negative correlation with Ni (Fig. 11c).

In the CS treatment, the Na levels in the stems were significantly negatively correlated with Cu, K, Ca, Mg, P, S, and B and significantly positively correlated with Mo, Zn, Mn, Al, Fe, Co, Se, and Si, but had a significantly negative correlation with Ni (Fig. 12a). In the SS treatment, the Na levels in the stems were significantly negatively correlated with Cu, Mg, P, Ca, and Se and significantly positively correlated with Fe, Al, Mn, Mo, Zn, S, Co, Si, and B, but had significantly negative correlations with K and Ni (Fig. 12b). In the AS treatment, the Na levels in the stems were significantly negatively correlated with Ca, P, Al, Cu, Mg, S, Fe, Se, and K and significantly positively correlated with Co, Mn, Mo, Zn, Ni, B, and Si (Fig. 12c).

In the CS treatment, the Na levels in the roots were negatively correlated with Ca, K, Fe, Ni, B, and Se and significantly positively correlated with Mg, Zn, Mo, Co, Mn, Cu, P, Al, S, and Si (Fig. 13a). In the SS treatment, the Na levels were significantly positively correlated with almost all elements, with Ca, P, and Se the exceptions (Fig. 13b). In the AS treatment, the Na levels in the roots were negatively correlated with P, Al, Fe, S, Se, Ca, and Zn and positively correlated with Mg, Mo, Mn, B, Co, Ni, K, Cu, and Si (Fig. 13c).

### Changes in expression of *GhSOS1* and *GhNHX1* after salt and alkali stresses

**Figure 14:**
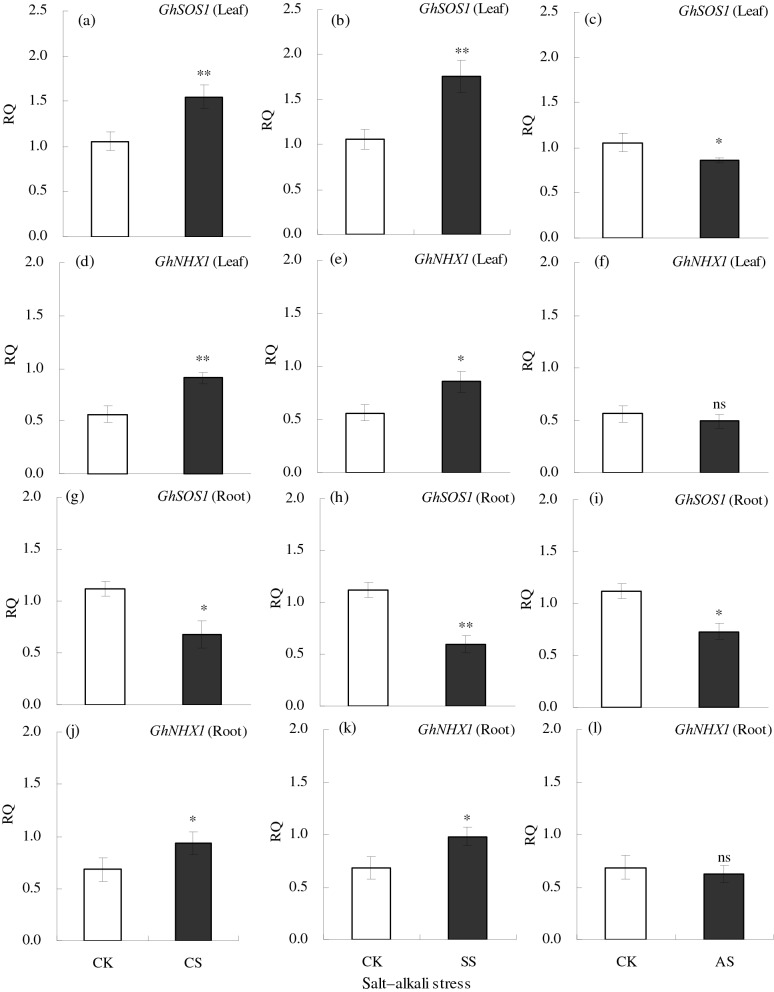
Relative expression (RQ) of the genes *GhSOS1* and *GhNHX1* in leaf and root of cotton under different types of salt–alkali stress (NaCl (CS), Na_2_SO_4_ (SS), and Na_2_CO_3_ + NaHCO_3_ (AS)). Columns with bars represent the mean ± standard error (*n* = 3). Asterisks indicate a significant difference between the control (CK) and the salt–alkali stress (*p < 0.05; **p < 0.01), and ns indicates no significant difference. (**a**–**c**) indicate the *GhSOS1* gene relative expression in cotton leaf under NaCl, Na_2_SO_4_, and Na_2_CO_3_ + NaHCO_3_ stress, respectively. (**d**–**f**) indicate the *GhNHX1* gene relative expression in cotton leaf under NaCl, Na_2_SO_4_, and Na_2_CO_3_ + NaHCO_3_ stress, respectively. (**g**–**i**) indicate the *GhSOS1* gene relative expression in cotton root under NaCl, Na_2_SO_4_, and Na_2_CO_3_ + NaHCO_3_ stress, respectively. (**j**–**l**) indicate the *GhNHX1* gene relative expression in cotton root under NaCl, Na_2_SO_4_, and Na_2_CO_3_ + NaHCO_3_ stress, respectively.

To determine the changes in Na^+^ transport-related genes of cotton under salt–alkali stress, the expression patterns of *GhSOS1* and *GhNHX1* were analyzed by RT-qPCR (Fig. 14). In the leaf, the expression of *GhSOS1* increased significantly under salt stress but decreased significantly under alkali stress. However, in the roots, the expression levels of *GhSOS1* decreased significantly in all salt–alkali treatments. The expression of *GhNHX1* increased significantly in leaves and roots under salt stress treatments; however, the difference between the CK and AS treatments was not significant for either leaves or roots.

## Discussion

### Effects of salt and alkali stresses on cotton growth

Excessive soil salinity mainly causes damage to plants through osmotic stress and ion toxicity, and the inhibition of growth is the most common physiological response in a saline habitat^25–27^. In this study, salt–alkali stress significantly inhibited cotton growth. The inhibition might have been due to the toxicity of Na ions with salt stress^28,29^ and the increase in pH and disturbance of plant nutrition and metabolism with alkali stress^30^. In addition, salt–alkali stress reduced root length, surface area, and root volume in this study. Chachar et al.^12^ found similar results in cotton, with salt stress inhibiting the elongation of main roots and the occurrence of lateral roots.

### Effects of salt and alkali stress on cotton physiological response

The structure and function of cell membranes are important in plant adaptability to adversity. Plants respond to salt stress by up-regulating protective enzymes, such as SOD, POD, and CAT, to increase the ability to scavenge reactive oxygen species (ROS)^31–34^. Under normal physiological conditions in plants, the continuous production and consumption of ROS are maintained in dynamic equilibrium, whereas salt stress destroys that dynamic equilibrium, resulting in the peroxidation and deacylation of membrane lipids. With such damage to the membrane system and metabolic processes, as well as to biological macromolecules such as proteins and nucleic acids, the result is cell death^35,36^. In this study, salt–alkali stress significantly increased the REC of leaves. In addition, the REC under alkali stress was significantly lower than that under salt stress, indicating that salt stress was more harmful to the permeability of leaf cell membranes. By contrast, the MDA content was significantly higher under alkali stress than under salt stress, with the lowest MDA content under Na_2_SO_4_ stress, indicating greater damage by ROS under alkali stress. Chen et al.^37^ also found that with increases in soil salinity and pH, both MDA content and REC increased significantly, especially at high soil salinity. Increasing the activity of antioxidant enzymes and the level of antioxidant metabolism is an important mechanism to improve the salt tolerance of plants. Moreover, SOD, POD, and CAT stabilize the ability to scavenge active oxygen-free radicals^38^. In this study, salt–alkali stress increased the activities of SOD, POD, and CAT in leaves. With a significant increase in SOD activity, the ability of leaves to scavenge oxygen-free radicals can increase, whereas with a significant increase in CAT activity, H_2_O_2_ can be eliminated by directly decomposing H_2_O_2_ into water and oxygen^39^. Zhang et al.^40^ and Ibrahim et al.^41^ also reported that salinity stress increases the activities of SOD, POD, and CAT in cotton leaves.

To protect themselves under salt stress, plants can also biosynthesize soluble compounds, such as carbohydrates, proline, and betaine, to adjust cellular osmotic conditions, maintain membrane integrity and function^42,43^, and stabilize enzyme activities. In addition, the increase in soil pH under alkali stress can inhibit the uptake of mineral elements by crops, and therefore, some crops secrete organic acids to activate mineral elements around the roots and resist the alkali stress. Proline is one of the organic acids, and its content might be higher under alkali stress. In this study, the Pro content was higher under alkali stress than under salt stress, which suggested that Pro played a more important role in resisting alkali stress. Several studies also show that Pro content increases with increasing soil salinity^39,44,45^.

### Effects of salt and alkali stresses on cotton tissue ionomes

The absorption of nutrients by plants is inhibited by salt–alkali stress^46,47^, and the lack of a particular nutrient element changes the metabolism of plants and affects the biosynthesis of metabolites. Salt stress is primarily caused by excess salt ions in soil, and rebuilding ion homeostasis under salt–alkali stress remains an important salt-tolerance strategy in plant cells^13,48^. Chen et al.^37^ suggest that osmotic adjustment by accumulating and absorbing inorganic ions such as Na^+^, K^+^, and Ca^2+^ in cells is an important salt-tolerance mechanism in plants; however, this adjustment can easily destroy the ion balance in cells and cause ion poisoning. Whether such ion poisoning will cause changes in other ions has become a focus of research. Wu et al.^16^ report that salt stress inhibits the uptake of macro and trace elements by crops, which leads to nutrient deficiency and cell metabolic disorder. In our experiment, the changes in ion groups in cotton under the three different types of salt–alkali stress were examined, and a total of 17 related ions were screened. According to PCAs, the elements that most strongly contributed to the first principal component were Na, Ca, Mn, Fe, Mo, Al, and Co in the leaf ionome; Na, P, Ca, Cu, Zn, and Mo in the stem ionome; and Na, P, Mg, Mn, Mo, and B in the root ionome.

In this study, salt–alkali stress increased the Na concentration in roots, stems, and leaves, with the concentration higher in leaves than in stems and roots, indicating that cotton could not prevent the transport of Na from the roots to the leaves. Consistent with the results of this study, Yang et al.^30^ also show that salt stress (NaCl, Na_2_SO_4_) and alkali stress (NaHCO_3_, Na_2_CO_3_) increase the Na concentration in plants. In addition, we found that the increase in Na concentration was higher in leaves, stems, and roots under alkali stress than under salt stress. Wang et al.^49^ also observed that the Na^+^ content is greater under alkali stress than under salt stress. Because more Na accumulates in the shoots than in the roots, the shoots are more sensitive to salt stress^50,51^. Cotton responds to salt stress by maintaining the balance of K and Na ions in tissues, and maintaining a relatively high K/Na ratio in tissues is more important than simply maintaining a lower Na concentration^52^. In this study, salt–alkali stress significantly decreased the K/Na ratio in cotton. The K/Na ratio was higher under alkali stress than under salt stress. Potassium is an essential nutrient element for plants, and when the Na concentration is higher than the K concentration in the external environment, Na ions inhibit K uptake by plants through competitive action^53^. Sodium can replace K at binding sites and thus inhibit the normal metabolism of plants. In this study, the leaf K concentration decreased under NaCl stress and alkali stress, but Na_2_SO_4_ and alkali stress had no significant effect on K absorption. This result might be attributed to an inhibitory effect of high pH on K transport, which relies on a transmembrane proton gradient^49^. Wang et al.^11^ also observed that salt stress significantly reduces the K concentration in cotton shoots and roots.

Salt–alkali stress inhibited P absorption in leaves, stems, and roots, which indicated that high-salt and high-pH environments inhibit P absorption and transportation in cotton. The most likely explanations for these results are the following: (1) under salt stress, the high concentration of Na^+^ and Cl^−^ in plants results in a significant decrease in the concentrations of K^+^, Ca^2+^, and P in plant leaves and roots, which hinders normal cell metabolism and material transportation^54^; and (2) under alkali stress, with high soil pH, Na^+^, Cl^−^, and CO_3_^2+^ ions compete with available P and exchangeable calcium combines with P to form insoluble calcium phosphate salts, which result in a sharp decrease in the concentration of available P in the rhizosphere of crops^55,56^. Sulfur is a component of amino acids in enzymes and other proteins and is involved in the formation of chlorophyll and the metabolism of carbohydrates. In this study, under NaCl and alkali stresses, the concentration of S in leaves decreased, but under Na_2_SO_4_ stress, the concentration of S increased significantly in roots, stems, and leaves, most likely because of the addition of SO_4_^2−^. In addition to K, Ca and Mg are also important in improving the salt tolerance of cotton^57,58^. Calcium and Na interact antagonistically, and excessive Na intake leads to a relative deficiency of Ca in cotton. The findings in this study are similar to those of Zhang et al.^59^ who reported that salt stress significantly decreases the Ca concentration in plants. However, Severino et al.^60^ showed that Ca and Mg do not reduce Na toxicity at the seedling stage of cotton. In this study, salt–alkali stress significantly increased the Mg concentration in roots but significantly decreased the concentration in leaves, indicating that salt–alkali stress inhibited the transport of Mg. The significant decrease in the Mg concentration in leaves might be due to lower chlorophyll concentration in the leaves after salt stress^59^.

The essential micronutrient elements in plants are Fe, Mn, Cu, Zn, B, and Mo, which are components of enzymes or coenzymes in plants, with strong specificity. Competition and interaction between soluble salts and mineral nutrients can lead to nutritional disequilibrium and deficiency^61^. Iron is an essential component in chlorophyll biosynthesis and respiration and is associated with enzymes. In this study, salt–alkali stress significantly increased the Fe concentration in leaves. The increase might be because cotton responded to salt–alkali stress by biosynthesizing chlorophyll in order to increase photosynthesis and maintain growth. Manganese has a catalytic role in chlorophyll biosynthesis, which is closely related to photosynthesis and respiration in plants. In our study, salt–alkali stress increased the Mn concentration in cotton plants. However, Karimi et al.^62^ reported that excessive accumulation of Na reduces the absorption of Mn. Copper is a component of some proteins in plants and participates in photosynthesis, in addition to increasing the stability of chloroplasts. In this study, both salt and alkali stress increased the Cu concentration in roots. However, salt stress decreased the Cu concentration in leaves, whereas alkali stress increased the Cu concentration in the leaves. Zinc is involved in the biosynthesis of auxins, and with a lack of Zn, crop growth and development are inhibited. To maintain their growth in a stressed environment, plants can promote the absorption of Zn. In this study, neutral salt stress significantly increased the Zn concentration in root, stem, and leaf, whereas alkali stress significantly increased the Zn concentration in stem and leaf. These results showed that salt stress promoted the absorption and transport of Zn, whereas alkali stress only promoted the transport of Zn to the shoots. Boron promotes carbohydrate transport and metabolism. In this study, Na_2_SO_4_ stress and alkali stress increased the B concentration in roots but decreased the concentration in leaves. However, NaCl stress showed the opposite effect. Molybdenum is the main component of nitrate reductase, which directly affects nitrogen metabolism. In this study, the Mo concentration in leaves, stems, and roots of cotton increased significantly under the different types of salt–alkali stress and was also significantly positively correlated with the Na concentration. Tang et al.^63^ found that the application of Mo can increase the salt tolerance of cotton, with the effectiveness of Mo increasing as the soil pH increased. Therefore, Mo can affect the salt and alkali resistance of cotton. We also found that alkali stress significantly increased the concentration of Ni in cotton roots, stems, and leaves, indicating that alkali stress promoted the absorption and transport of Ni, suggesting it may play an important role in cotton resistance to alkali stress. Silicon, Co, Se, and Al are beneficial elements, and changes in their concentrations are caused by changes in other ions, which are companion ions and are not associated with salt and alkali resistance in plants.

On the basis of the comparative analysis of the three ionomes in cotton under salt–alkali stress, the inhibition of ion absorption was greater under alkali stress than under salt stress, because the concentrations of more elements were reduced in roots, stems, and leaves under alkali stress than under neutral salt stress. Therefore, under neutral salt stress, the absorption of only individual ions decreased, and as a result, neutral salt stress mainly disrupted the ion balance. However, alkali stress also inhibited the absorption of mineral elements, in addition to disrupting the ion balance.

### Effects of salt and alkali stresses on expression of *GhSOS1* and *GhNHX1*

The key ion that affects the ion homeostasis of cotton under saline–alkali stress is Na^+^. Therefore, it is critical to study the molecular mechanisms involving Na^+^ to better understand the changes in ion homeostasis. *GhSOS1* and *GhNHX1* are two key salt-tolerance genes of cotton. *GhSOS1* mainly regulates the plasma membrane Na^+^/H^+^antiporter, which can exclude excess Na^+^ from the cytoplasm^19,64^. In our study, the relative expression of *GhSOS1* increased significantly in cotton leaves and roots under neutral salt (NaCl and Na_2_SO_4_) stress, but under alkali stress, the relative expression decreased significantly, indicating that high pH could inhibit *GhSOS1* expression in leaf cells. This result might explain the different ion changes in cotton and was also consistent with the results for Na in the cotton ionome under alkali stress. *GhNHX1* is a vacuolar membrane-bound Na^+^/H^+^ antiporter, which can transport Na^+^ from the cytoplasm into vacuoles and reduce the toxicity of excess Na^+^ in the cytoplasm, thereby regulating ion homeostasis in cotton^20,65^. In our study, salt stress significantly increased the relative expression of *GhNHX1* in roots and leaves. The up-regulation of *GhNHX1* under salt stress could help cotton to isolate excess Na^+^ and regulate ion homeostasis. In addition, alkali stress reduced the relative expression of *GhNHX1* somewhat in this study, but the effect was not significant. It may be that when the degree of alkali stress reaches a critical level in cotton growth, the salt tolerance mechanisms are destroyed, including a decline in *GhNHX1* expression. In previous studies, the overexpression of *SOS1*^66^ or *NHX1*^67^ in transgenic plants increases salt tolerance.

## Conclusions

Salt–alkali stress inhibited cotton growth and reduced root length, surface area, and volume, and the K/Na ratio of cotton, but increased REC, MDA, PRO content and antioxidant enzyme activities. Alkali stress inhibited ion absorption more than salt stress. Although neutral salt stress mainly disrupted the ion balance, alkali stress also inhibited the absorption of mineral elements, in addition to disrupting the ion balance. Cotton can adapt to salt–alkali stress through the formation of new ionic homeostasis. However, strategies differ in cotton under salt–alkali stress. Under NaCl stress, the absorption of Ca in cotton is inhibited, the transport capacity of P, Mg, and Cu is reduced, and the ion balance is maintained by promoting the absorption and transport of Zn, Mn, Al, and Mo; Under Na_2_SO_4_ stress, the absorption of P and Ca in cotton is inhibited, the transport capacity of Mg, B, and Cu is reduced, and the ion balance is maintained by promoting the uptake and transport of S, Zn, Fe, Mo, Al, and Co; Under Na_2_CO_3_ + NaHCO_3_ stress, the absorption of P and S in cotton is inhibited, the transport capacity of Mg and B is reduced, and the ion balance is maintained by promoting the absorption and transport of Mn, Mo, Ni, and Co. In addition, the relative expression of *GhSOS1* and *GhNHX1* in leaves decreased under Na_2_CO_3_ + NaHCO_3_ stress but increased significantly under NaCl and Na_2_SO_4_ stress. The changes in the expression of *GhSOS1* and *GhNHX1* might partially explain the accumulation of Na ions under different types of salt–alkali stress in cotton.

## Materials and methods

### Materials

The experiment was conducted in a greenhouse at the experimental station (N 44°18′, E 86°02′) of the College of Agriculture, Shihezi University, China, in 2019. The soil used in the experiment was collected from the 0 to 30-cm depth at the experimental station. The soil type was grey desert soil with a loam texture, and the basic soil properties were as follow: soil salinity, 0.35 dS m^−1^; pH, 7.86; total nitrogen, 0.58 g kg^−1^; organic matter, 9.45 g kg^−1^; available phosphorus, 6.71 mg kg^−1^; available potassium, 142 mg kg^−1^. The cotton cultivar was Lu-mian-yan No. 24.

### Experimental design

Three common types of soil salinization were tested in the experiment, including chloride (NaCl, CS), sulfate (Na_2_SO_4_, SS), and carbonate (Na_2_CO_3_ + NaHCO_3_, AS). Non-salt-alkali stress was set as the control. There were three replicates of each treatment. The specific experimental treatments, i.e., soil salinization types, and their salinization degree are shown in Table 1.

**Table 1:** Type and degree of saline and alkaline stress in the soils of different treatments.

Treatment	Salinity and alkalinity	EC_1:5_ (dS m^−1^)	pH (1:2.5)
Control (CK)	No salinization or alkalization	0.35	8.16
NaCl (CS)	Moderate salinization	1.39	8.43
Na_2_SO_4_ (SS)	Moderate salinization	2.01	8.19
Na_2_CO_3_ + NaHCO_3_ (AS)	Moderate alkalization	0.63	9.92

Before initiating the experiment, the field-collected soil was naturally dried, crushed, and passed through a 2-mm sieve. Then, the solutions of NaCl, Na_2_SO_4_, or Na_2_CO_3_ + NaHCO_3_ (weight ratio 1:1) at different concentrations were added to the soil to produce a supersaturated state (the same volume of deionized water was added to the control soil) for 1 month to achieve the equilibrium of the soil. Thus, three different types of salt soils were formed. Then, the three types of saline soil were naturally dried, crushed, and passed through a 2-mm sieve. Soil columns that were 20 cm in diameter and 60 cm in height were prepared. The soil was layered to the 50-cm depth at the soil bulk density of 1.25 g cm^−3^, with 10 cm per layer and 20 kg per soil column. The columns were drip-irrigated, and the emitters (columns) were 0.4 m apart with a discharge rate (pressure compensated) of 2.1 L h^−1^. The drip irrigation pipe was laid flat on the surface of the soil columns, with each soil column supplied by one emitter fixed at the center of the top of the column.

Cotton was sowed on 28 April 2019, and 10 seeds were sown per soil column. To ensure cotton emergence, each soil column was irrigated with 3 L of water after sowing. When the cotton seedlings reached the “2 leaves and 1 heart” stage, two cotton seedlings with uniform growth were retained in each soil column. To ensure an adequate water supply, water was replenished by drip irrigation at regular intervals during the experiment to maintain the soil moisture content at 60% to 80% of field capacity. The experiment ended 60 days after sowing.

### Sample collection and treatment

#### Growth

To determine the dry matter of cotton, three representative cotton plants were selected in each treatment. The roots, stems, and leaves were separated in the laboratory, and the fresh material was heat-treated at 105 °C for 30 min. Then, the materials were oven-dried at 70 °C for 48 h, weighed, ground to pass through a 1-mm sieve, and stored at room temperature.

#### Root morphology

To collect the roots, a soil column (soil + root system) was put in a nylon net, and the soil was washed away in running water. The intact root system was removed and stored in a ziplock bag in an ultra-low temperature refrigerator. Roots were scanned with a flatbed image scanner (Epson Expression 1600 scanner). To determine root length, surface area, and volume, the images were analyzed using WinRhizo software (V5.0, Regent Instruments, Quebec, Canada).

### Relative electrical conductivity, malondialdehyde, and antioxidant enzyme activity

Sixty days after cotton seedling emergence, all functional leaves on the main stem (the third leaves on the main stem were completely unfolded) were collected in each treatment and then transported to the laboratory in an icebox. The dust and dirt on leaf surfaces were removed; the moisture on the surface was wiped with an absorbent paper; and the main vein was removed. The relative electrical conductivity (REC) of leaves was measured by the conductance method. The malondialdehyde (MDA) content in leaves was measured according to Wu et al.^68^, and the proline (Pro) content was measured according to Bates et al.^69^. The SOD activity was measured according to Zhang et al.^70^, the POD activity according to Tan et al.^71^, and the CAT activity according to Cakmak and Marschner^72^.

### Ionomes

The plant ionomic analysis included the following steps. The leaves, stems, and roots were crushed and passed through sieves. Then, 10 mL of concentrated nitric acid was added to 100 mg of each sample, which was followed by digestion in a microwave digestion instrument (Milestone, ETHOSA). After microwave digestion, the samples were placed on an electric heating plate at 230 °C for approximately 20 min to drive off the acid. After the digestion tank was removed, the solution was transferred to a 25-mL colorimetric tube with ultrapure water. The microwave digestion tank and the lid were rinsed 3 to 5 times to remove all materials. A buffer solution was transferred to the colorimetric tube, diluted to volume, and then shaken evenly. The ion concentrations (Na, P, K, Ca, Mg, S, Fe, B, Mn, Zn, Cu, Mo, Ni, Si, Co, Al, and Se) in leaves, stems, and roots were measured using inductively coupled plasma mass spectrometry (Agilent 7700X ICP-MS, USA).

### Gene expression

The expression of *GhSOS1* and *GhNHX1* was assessed using reverse-transcription quantitative PCR (qRT-PCR).The primers used in the RT-qPCR analysis are listed in Table 2. The qRT-PCR method is given by Peng et al.^73^. The qRT-PCR was performed on an ABI PRISM 7300 Sequence Detection System using 0.1 μL of cDNA, 5 μL of SYBR Premix Ex Taq II (Takara, Dalian, China), 0.4 μL of each primer (forward and reverse, 10 μmol/L), and H_2_O added to a final reaction volume of 10 μL. The qPCR conditions were as follows: preincubation at 95 °C for 5 min, followed by 40 cycles at 95 °C for 15 s and 60 °C for 1 min. The UBQ7 was chosen as the housekeeping gene for standardizing the qRT-PCR experiments. The relative expression level of each gene was determined relative to UBQ7 as a housekeeping gene and was calculated using the 2^−ΔΔCT^ method^74^.

**Table 2:** Primers used to determine gene expression in reverse-transcription quantitative PCR.

Gene name	Annotation	Sequence 5′–3′
*UBQ7*		F: GAAGGCATTCCACCTGACCAAC
		R: CTTGACCTTCTTCTTCTTGTGCTTG
*GhSOS1*	Plasma membrane Na^+^/H^+^ antiporter	F: AGTGTCAGCCAATAAACAACC
		R: TCTTTCGTGTCCATCTTCTTC
*GhNHX1*	Tonoplast Na^+^/H^+^ antiporter	F: TTCTCTTTCTTTATGTCGGGATG
		R: AACAAGACCCATCAGCACAGC

### Data analysis

The data were analyzed using SPSS 21.0 software (SPSS Inc., Chicago, IL, USA). Values are presented as the mean (*n* = 3) ± standard error (SE). Duncan’s multiple range tests were conducted to determine whether there were significant differences between individual treatments at *p* < 0.05. Pearson’s correlation analyses were performed between the concentrations of Na and those of other minerals in the different tissues (*p* < 0.05). The R package software (Version 4.0.3) was used for principal component analysis (PCA) of the ionome. For hierarchical cluster analysis of the ionomes in cotton plants, the online software was used at MetaboAnalyst website (http://www.metaboanalyst.ca/).

## Supplementary Information

Supplementary Information.

## Data Availability

All data generated or analysed during this study are included in this published article (and its Supplementary Information files).
