# Supplementary material for: Growth, ionic homeostasis, and physiological responses of cotton under different salt and alkali stresses
Source: Sci Rep. 2020 Dec 14;10:21844. doi: 10.1038/s41598-020-79045-z (PMC7736318; doi:10.1038/s41598-020-79045-z)
Supplement: Supplementary file 1 — Supplementary Information. [file 41598_2020_79045_MOESM1_ESM.doc]

| Treatment | Na/ppm | P/ppm | K/% | Ca/% | Mg/% | S/% | Fe/ppm | Mn/ppm | Zn/ppm | Cu/ppm | B/ppm | Mo/ppm | Ni/ppm | Co/ppm | Al/ppm | Si/ppm | Se/ppm |
| --- | --- | --- | --- | --- | --- | --- | --- | --- | --- | --- | --- | --- | --- | --- | --- | --- | --- |
| CK | 1120 | 2767 | 2.67 | 5.67 | 1.17 | 2.10 | 238 | 151 | 58.0 | 7.69 | 197 | 4.41 | 0.52 | 0.09 | 307 | 246 | 0.10 |
| CS | 7434** | 2217* | 2.23* | 4.45** | 0.95* | 1.80* | 299** | 207** | 76.6** | 6.83** | 208* | 5.35** | 0.49ns | 0.13* | 470** | 208* | 0.09ns |

**Table S1.** Effects of NaCl stress on the concertration of mineral elements in leaves of cotton

Note: Asterisks indicate a significant difference between the control (CK) and the salt–alkali stress (**p < 0.05***, *p < 0.01*), and ns indicates no significant difference.

**Table S2. Effects of Na_2_SO_4_ stress on the concertration of mineral elements in leaves of cotton**

| Treatment | | Na/ppm | | P/ppm | | K/% | | Ca/% | | Mg/% | | S/% | | Fe/ppm | | Mn/ppm | | Zn/ppm | | Cu/ppm | | B/ppm | Mo/ppm | Ni/ppm | Co/ppm | Al/ppm | Si/ppm | Se/ppm |
| --- | --- | --- | --- | --- | --- | --- | --- | --- | --- | --- | --- | --- | --- | --- | --- | --- | --- | --- | --- | --- | --- | --- | --- | --- | --- | --- | --- | --- |
| CK | 1121 | | 2767 | | 2.67 | | 5.67 | | 1.17 | | 2.10 | | 238 | | 151 | | 58.0 | | 7.69 | | 197 | | 4.41 | 0.52 | 0.09 | 307 | 246 | 0.10 |
| SS | 15537** | | 2064** | | 2.75ns | | 4.19** | | 1.01* | | 2.78** | | 288** | | 154* | | 90.7** | | 7.49* | | 185** | | 8.62** | 0.51ns | 0.11* | 439** | 2038* | 0.04* |

Note: Asterisks indicate a significant difference between the control (CK) and the salt–alkali stress (**p < 0.05***, *p < 0.01*), and ns indicates no significant difference.

**Table S3. Effects of Na_2_CO_3_ + NaHCO_3_ stress on the concertration of mineral elements in leaves of cotton**

| Treatment | | Na/ppm | | P/ppm | | K/% | | Ca/% | | Mg/% | | S/% | | Fe/ppm | | Mn/ppm | | Zn/ppm | | Cu/ppm | | B/ppm | Mo/ppm | Ni/ppm | Co/ppm | Al/ppm | Si/ppm | Se/ppm |
| --- | --- | --- | --- | --- | --- | --- | --- | --- | --- | --- | --- | --- | --- | --- | --- | --- | --- | --- | --- | --- | --- | --- | --- | --- | --- | --- | --- | --- |
| CK | 1121 | | 2767 | | 2.67 | | 5.67 | | 1.17 | | 2.10 | | 238 | | 151 | | 58.0 | | 7.69 | | 197 | | 4.41 | 0.52 | 0.09 | 307 | 246 | 0.10 |
| AS | 20856** | | 2383* | | 2.39* | | 3.40* | | 0.85** | | 1.73** | | 408** | | 274** | | 69.4** | | 8.48** | | 157* | | 10.32** | 0.86* | 0.19** | 619** | 3552* | 0.05* |

Note: Asterisks indicate a significant difference between the control (CK) and the salt–alkali stress (**p < 0.05***, *p < 0.01*), and ns indicates no significant difference.

**Table S4. Effects of NaCl stress on the concertration of mineral elements in stem of cotton**

| Treatment | | Na/ppm | | P/ppm | | K/% | | Ca/% | | Mg/% | | S/% | | Fe/ppm | | Mn/ppm | | Zn/ppm | | Cu/ppm | | B/ppm | | Mo/ppm | | Ni/ppm | | Co/ppm | | Al/ppm | | Si/ppm | | Se/ppm |
| --- | --- | --- | --- | --- | --- | --- | --- | --- | --- | --- | --- | --- | --- | --- | --- | --- | --- | --- | --- | --- | --- | --- | --- | --- | --- | --- | --- | --- | --- | --- | --- | --- | --- | --- |
| CK | 714 | | 2091 | | 2.17 | | 1.41 | | 0.59 | | 0.28 | | 73.1 | | 13.8 | | 25.6 | | 7.03 | | 21.3 | | 1.60 | | 0.47 | | 0.06 | | 75.1 | | 56.0 | | 0.025 | |
| CS | 3451** | | 1917* | | 1.66** | | 1.04** | | 0.47** | | 0.26* | | 93.6 | | 19.6** | | 34.9** | | 5.86** | | 18.8* | | 2.51** | | 1.01ns | | 0.10* | | 99.4** | | 67.1ns | | 0.033ns | |

Note: Asterisks indicate a significant difference between the control (CK) and the salt–alkali stress (**p < 0.05***, *p < 0.01*), and ns indicates no significant difference.

**Table S5.** Effects of Na_2_SO_4_ stress on the concertration of mineral elements in stem of cotton

| Treatment | | Na/ppm | | P/ppm | | K/% | | Ca/% | | Mg/% | | S/% | | Fe/ppm | | Mn/ppm | | Zn/ppm | | Cu/ppm | | B/ppm | | Mo/ppm | | Ni/ppm | | Co/ppm | | Al/ppm | | Si/ppm | Se/ppm |
| --- | --- | --- | --- | --- | --- | --- | --- | --- | --- | --- | --- | --- | --- | --- | --- | --- | --- | --- | --- | --- | --- | --- | --- | --- | --- | --- | --- | --- | --- | --- | --- | --- | --- |
| CK | 714 | | 2091 | | 2.17 | | 1.41 | | 0.59 | | 0.28 | | 73.1 | | 13.8 | | 25.6 | | 7.03 | | 21.3 | | 1.60 | | 0.47 | | 0.06 | | 75.1 | | 56.0 | | 0.025 |
| SS | 9192** | | 1571** | | 2.17ns | | 0.94** | | 0.49** | | 0.33** | | 139** | | 19.0** | | 33.9** | | 5.00** | | 22.5ns | | 2.13** | | 0.47ns | | 0.08* | | 214** | | 78.9ns | | 0.016* |

Note: Asterisks indicate a significant difference between the control (CK) and the salt–alkali stress (**p < 0.05***, *p < 0.01*), and ns indicates no significant difference.

**Table S6. Effects of Na_2_CO_3_ + NaHCO_3_ stress on the concertration of mineral elements in stem of cotton**

| Treatment | Na/ppm | P/ppm | K/% | Ca/% | Mg/% | S/% | Fe/ppm | Mn/ppm | Zn/ppm | Cu/ppm | B/ppm | Mo/ppm | Ni/ppm | Co/ppm | Al/ppm | Si/ppm | Se/ppm |
| --- | --- | --- | --- | --- | --- | --- | --- | --- | --- | --- | --- | --- | --- | --- | --- | --- | --- |
| CK | 714 | 2091 | 2.17 | 1.41 | 0.59 | 0.28 | 73.1 | 13.8 | 25.6 | 7.03 | 21.3 | 1.60 | 0.47 | 0.06 | 75.1 | 56.0 | 0.025 |
| AS | 12733** | 1741** | 2.15ns | 0.99** | 0.53** | 0.24** | 65.4* | 29.4** | 32.4** | 5.88** | 25.3* | 2.27** | 0.65* | 0.10ns | 63.2** | 353* | 0.017ns |

Note: Asterisks indicate a significant difference between the control (CK) and the salt–alkali stress (**p < 0.05***, *p < 0.01*), and ns indicates no significant difference.

**Table S7.** Effects of NaCl stress on the concertration of mineral elements in root of cotton

| Treatment | | Na/ppm | | P/ppm | | K/% | | Ca/% | | Mg/% | | S/% | | Fe/ppm | | Mn/ppm | | Zn/ppm | | Cu/ppm | | B/ppm | | Mo/ppm | | Ni/ppm | | Co/ppm | | Al/ppm | | Si/ppm | | Se/ppm |
| --- | --- | --- | --- | --- | --- | --- | --- | --- | --- | --- | --- | --- | --- | --- | --- | --- | --- | --- | --- | --- | --- | --- | --- | --- | --- | --- | --- | --- | --- | --- | --- | --- | --- | --- |
| CK | 504 | | 1357 | | 1.02 | | 0.38 | | 0.17 | | 0.12 | | 263 | | 9.1 | | 15.4 | | 3.28 | | 10.3 | | 1.29 | | 0.45 | | 0.12 | | 347 | | 32.6 | | 0.022 | |
| CS | 2103** | | 1518* | | 0.98ns | | 0.33* | | 0.18ns | | 0.12ns | | 256ns | | 12.1* | | 23.6** | | 3.42* | | 10.1ns | | 1.73** | | 0.44ns | | 0.17* | | 389* | | 41.9ns | | 0.022ns | |

Note: Asterisks indicate a significant difference between the control (CK) and the salt–alkali stress (**p < 0.05***, *p < 0.01*), and ns indicates no significant difference.

**Table S8.** Effects of Na_2_SO_4_ stress on the concertration of mineral elements in root of cotton

| Treatment | | Na/ppm | | P/ppm | | K/% | | Ca/% | | Mg/% | | S/% | | Fe/ppm | | Mn/ppm | | Zn/ppm | | Cu/ppm | | B/ppm | | Mo/ppm | | Ni/ppm | | Co/ppm | | Al/ppm | | Si/ppm | | Se/ppm |
| --- | --- | --- | --- | --- | --- | --- | --- | --- | --- | --- | --- | --- | --- | --- | --- | --- | --- | --- | --- | --- | --- | --- | --- | --- | --- | --- | --- | --- | --- | --- | --- | --- | --- | --- |
| CK | 504 | | 1357 | | 1.02 | | 0.38 | | 0.17 | | 0.12 | | 263 | | 9.1 | | 15.4 | | 3.28 | | 10.3 | | 1.29 | | 0.45 | | 0.12 | | 347 | | 32.6 | | 0.022 | |
| SS | 3380** | | 1061** | | 1.06ns | | 0.31** | | 0.21ns | | 0.14* | | 337** | | 11.3** | | 21.1** | | 3.43* | | 10.9* | | 1.80** | | 0.53* | | 0.17** | | 423** | | 44.9ns | | 0.019ns | |

Note: Asterisks indicate a significant difference between the control (CK) and the salt–alkali stress (**p < 0.05***, *p < 0.01*), and ns indicates no significant difference.

**Table S9. Effects of Na_2_CO_3_ + NaHCO_3_stress on the concertration of mineral elements in root of cotton**

| Treatment | | Na/ppm | | P/ppm | | K/% | | Ca/% | | Mg/% | | S/% | | Fe/ppm | | Mn/ppm | | Zn/ppm | | Cu/ppm | | B/ppm | | Mo/ppm | | Ni/ppm | | Co/ppm | | Al/ppm | | Si/ppm | | Se/ppm |
| --- | --- | --- | --- | --- | --- | --- | --- | --- | --- | --- | --- | --- | --- | --- | --- | --- | --- | --- | --- | --- | --- | --- | --- | --- | --- | --- | --- | --- | --- | --- | --- | --- | --- | --- |
| CK | 504 | | 1357 | | 1.02 | | 0.38 | | 0.17 | | 0.12 | | 263 | | 9.1 | | 15.4 | | 3.28 | | 10.3 | | 1.29 | | 0.45 | | 0.12 | | 347 | | 32.6 | | 0.022 | |
| AS | 3466** | | 896*8 | | 1.06ns | | 0.38ns | | 0.23ns | | 0.10* | | 208* | | 16.6** | | 15.4ns | | 3.33ns | | 12.1* | | 1.97** | | 0.52* | | 0.15* | | 250** | | 43.6ns | | 0.019ns | |

Note: Asterisks indicate a significant difference between the control (CK) and the salt–alkali stress (**p < 0.05***, *p < 0.01*), and ns indicates no significant difference.
